# Dream Psychology

**Published:** 1919

**Authors:** 


**Dream Psychology.** By MAURICE NICOLL, B.A., M.B., B.C. Camb. Pp. ix., 194. London: Oxford Medical Publications. 1917. Price 6s. net.

The analysis of the dream was initiated by Dr. Hughlings Jackson as the key to many problems of psychiatry. Dr. John Abercrombie, Dr. W. B. Carpenter, and others have pointed out that the understanding of dreams is closely linked with the investigation of mind. The theories and demonstrations on cerebral localisation have brought into the forefront the anatomical considerations which have engrossed the attention of psychiatrists and neurologists, but of late physiological has rather taken the place of further anatomical inquiry. The Viennese school under Professor Freud, the pioneer of dream analysis, is being supplanted by the Zurich school of analytical psychology associated with the name of Dr. Jung, from whose point of view the author regards the dream and the interpretation thereof.

The dream is said to arise somewhere out of the psyche, a typical product of the unconscious regions of the human mind, and several schools of psychology are concerned in the study of these unconscious activities.

Nineteen short chapters on the psyche and the problems of the neurotic sum up the views of the author on unconscious cerebration and its phenomena around which so much controversy is accumulating. The author has made a somewhat intricate subject both intelligible and entertaining.

